# A New Discovery of Argon Functioning in Plants: Regulation of Salinity Tolerance

**DOI:** 10.3390/antiox11061168

**Published:** 2022-06-14

**Authors:** Jun Wang, Chenxu Cai, Puze Geng, Feng Tan, Qing Yang, Ren Wang, Wenbiao Shen

**Affiliations:** 1 College of Life Sciences, Nanjing Agricultural University, Nanjing 210095, China; 2020116099@stu.njau.edu.cn (J.W.); 2021116098@stu.njau.edu.cn (C.C.); 14819125@njau.edu.cn (P.G.); tanfeng@njau.edu.cn (F.T.); qyang19@njau.edu.cn (Q.Y.); 2 Institute of Botany, Jiangsu Province and Chinese Academy of Sciences, Nanjing 210014, China; rwang@cnbg.net

**Keywords:** alfalfa, argon gas, ion homeostasis, antioxidant defense, salinity tolerance

## Abstract

Argon, a non-polar molecule, easily diffuses into deeper tissue and interacts with larger proteins, protein cavities, or even receptors. Some of the biological effects of argon, notably its activity as an antioxidant, have been revealed in animals. However, whether and how argon influences plant physiology remains elusive. Here, we provide the first report that argon can enable plants to cope with salinity toxicity. Considering the convenience of the application, argon gas was dissolved into water (argon-rich water (ARW)) to investigate the argon’s functioning in phenotypes of alfalfa seed germination and seedling growth upon salinity stress. The biochemical evidence showed that NaCl-decreased *α*/*β*-amylase activities were abolished by the application of ARW. The qPCR experiments confirmed that ARW increased *NHX1* (Na^+^/H^+^ antiporter) transcript and decreased *SKOR* (responsible for root-to-shoot translocation of K^+^) mRNA abundance, the latter of which could be used to explain the lower net K^+^ efflux and higher K accumulation. Subsequent results using non-invasive micro-test technology showed that the argon-intensified net Na^+^ efflux and its reduced Na accumulation resulted in a lower Na^+^/K^+^ ratio. NaCl-triggered redox imbalance and oxidative stress were impaired by ARW, as confirmed by histochemical and confocal analyses, and increased antioxidant defense was also detected. Combined with the pot experiments in a greenhouse, the above results clearly demonstrated that argon can enable plants to cope with salinity toxicity via reestablishing ion and redox homeostasis. To our knowledge, this is the first report to address the function of argon in plant physiology, and together these findings might open a new window for the study of argon biology in plant kingdoms.

## 1. Introduction

Saline soils can not only inhibit seed germination and seedling growth, but also reduce crop yields. Normally, sodium chloride (NaCl) is added to the culture medium to mimic salt stress, which is a combination of osmotic stress and ion (for example Na^+^) toxicity. Plants have developed several strategies to confront the challenges of sodium chloride salinity stress, including reestablishing ion and redox homeostasis [1]. The regulation of Na^+^ movement across the plasma membranes and tonoplast is an important strategy for plants adaptation to salt stress [2]. Since the inhibition of K^+^ uptake in salt-stressed plants also results in ion imbalance [3], maintaining higher levels in the K^+^ to Na^+^ ratio is important for plants to resist sodium chloride salinity stress [4].

Additionally, salinity stress can result in redox imbalance, which is usually confirmed by the overproduction of reactive oxygen species (ROS), including ^1^O_2_, H_2_O_2_, O_2_^−^, and HO [5]. Although some ROS have signaling effects, especially during the early period under stressed conditions, the final accumulation of ROS in cells can negatively influence plant metabolism, including the deactivation of enzymatic activities and the degradation of important proteins and genetic materials [6]. Accordingly, the detoxification of overproduction ROS has a vital role in plant tolerance against salinity stress.

Argon (Ar) is a one-atom, odorless, and extremely inert gas. It is the most common in the atmosphere at about 9340 ppm. Although called an inert gas, previous results have shown that argon might be a bioactive molecule that can be used for anesthesia or neuroprotection [7,8]. Since argon can easily diffuse into tissues, this gas can interact with amphiphilic proteins [9]. Subsequent work has shown that the neuroprotective function of argon is achieved by activating the transcription factor NF-E2-related factor, thus modulating the gene expression of antioxidant defense in animals [10]. Other effects were accomplished through the influence of the gamma-aminobutyric acid (GABA)_A_ receptor [11]. The participation of the phosphorylation of extracellular signal-regulated kinase (ERK)-1/2 and antioxidant machinery, including heme oxygenase-1 (HO-1), was also suggested [12,13,14]. For food preservation, a beneficial role for argon was reported for the postharvest quality and browning of sweet cherries [15] and fresh-cut apples at cold storage [16]. However, the requirement of higher pressure for argon limits its applications. Most importantly, it is unknown whether or how argon influences plant physiology, especially in response to different environmental stress conditions. Since argon not only exists abundantly in the atmosphere but also has therapeutic potential in medicine, we therefore deduced that this inert gas might have physiological functions in plants.

To investigate this scientific hypothesis, argon-rich water (ARW) was selected in this experiment to simulate the role of argon in plant biology, since this approach is easily controlled compared to argon gas. We first identified argon-facilitated tolerance against NaCl stress in laboratory and greenhouse conditions during alfalfa seed germination and seedling growth, two important development stages sensitive to NaCl stress [17]. The beneficial actions achieved by argon were positively correlated with the regulation of ion and redox homeostasis. Therefore, our findings shed new light on argon-based biology.

## 2. Materials and Methods

### 2.1. Preparation of Argon-Rich Water (ARW)

To prepare the ARW solution, argon gas with 99.99% purity (Nanjing Special Gases Factory Co., Ltd., Nanjing, China) was bubbled into 0.5 L distilled water at a speed of 200 mL min^−1^ for 50 min. This solution was regarded as the saturated stock solution (100% ARW; containing about 33.6 mL argon in 1 L solution). After dilution, the required concentrations of ARW (10%, 25%, and 50% saturation (*v/v*), equivalent to about 0.150, 0.375, and 0.750 mmol L^−1^) were obtained and used immediately.

### 2.2. Plant Materials, Growth Conditions, and Germination Experimental Treatments

Uniform alfalfa seeds (*Medicago sativa* L. “Victoria”) from Clover Company (Beijing, China), were transferred to Petri dishes and treated with 4 mL of distilled water, different concentrations of ARW, 100 mM NaCl alone, and various combinations of these treatments for 72 h in a growth chamber (25 ± 1 °C, 14 h photoperiod, and light intensity of 200 μmol m^−2^ s^−1^) [18]. These solutions were correspondingly renewed every 12 h. The control (Con) was treated with distilled water alone. Afterwards, the plants were photographed.

### 2.3. Germination and Growth Analysis

The germination experiment was implemented with three replicates, with 100 seeds per replicate. Each petri dish contained 50 seeds. Germination percentage (%) and root length (cm) were recorded at the indicated time points [19].

### 2.4. Pot Culture Experiment and Determination of Growth Parameters

After germinating for 7 days in distilled water, alfalfa plants were transplanted into pots (diameter × height, 8.5 cm × 8.0 cm; vermiculite: peat, 3:1, *v/v*). Three alfalfa plants were planted per pot. After growth in the greenhouse for 14 days, alfalfa plants were further subjected to further treatment with or without 100 mL of 50% ARW and simultaneously exposed to salinity stress by irrigating them with 100 mM NaCl (100 mL). Afterwards, all pots were irrigated with 100 mL distilled water every 2 days. After 21 days, representative individuals were photographed, and the phenotypic characteristics of nine plants, in terms of primary root length and fresh weight, were determined.

### 2.5. Measurement of Amylase Activity

Fresh alfalfa seedlings (0.5 g) were homogenized in distilled water. After centrifugation, supernatants were used as crude enzymes to analyze the amylase activity. *α*- and *β*-amylase activities were analyzed with the 3,5-dinitrosalicylic acid (DNS) method [20].

### 2.6. Analysis of Ion Fluxes with Non-Invasive Micro-Test Technology (NMT)

Net Na^+^ and K^+^ fluxes at the maturation zone of the alfalfa roots were monitored using NMT (BIO-IM NMT system, YoungerUSA LLC., Amherst, MA, USA), and calculated using imFluxes software (V3.0, Xuyue Company, Beijing, China) [21].

### 2.7. Determination of Ion Content

The Na^+^, K^+^, and Ca^2+^ contents of the alfalfa plants were determined according to a previously described method [22]. In brief, 0.1 g of dry sample was digested using a Microwave Digital Digestion System (Milestone Ethos T, Sorisole, Italy), and then the concentration of the ions above was measured with an inductively coupled plasma optical emission spectrometer (ICP-OES; Optima 8000, Perkin Elmer, Waltham, MA, USA).

### 2.8. Histochemical Staining

To analyze lipid peroxidation and plasma membrane integrity in the roots, Schiff’s reagent and Evans blue were used for staining [23]. The distribution of H_2_O_2_ or O_2_^−^ was visualized in the root tissues by separately staining them with 3,3-diaminobenzidine tetrahydrochloride (DAB) and nitroblue tetrazolium (NBT) [24]. For Schiff’s reagent staining, alfalfa root tissues were immersed in Schiff’s solution (0.5% *w/v*) and then incubated in darkness. For Evans blue staining, alfalfa root tissues were immersed in Evans blue solution (1% *w/v*) and then incubated in darkness. For DAB staining, alfalfa root tissues were immersed in DAB solution (1% *w/v*, pH 3.8) and then incubated in darkness. For NBT staining, alfalfa root tissues were immersed in NBT solution (0.1% *w/v* in 50 mM sodium phosphate buffer, pH 7.5) and incubated in darkness. After staining, root tissues were photographed (Stemi 2000-C; Carl Zeiss, Oberkochen, Germany).

### 2.9. Fluorescent-Based Histochemical Analysis

Free Na^+^ and reactive oxygen species (ROS) contents in the root tissues were visualized with specific fluorescent probes, including sodium-binding benzofuran isophthalate-AM (SBFI-AM) [25] and 2′,7′-dichlorofluorescein diacetate (DCFH-DA) [26], respectively.

The root tissues were visualized with a Zeiss LSM 800 confocal microscope (Carl Zeiss, Oberkochen, Germany) after dyeing them with fluorescent probes, using an excitation of 480 nm and emission of 500 nm for Na^+^ analysis and an excitation of 488 nm and emission of 525 nm for ROS analysis. The relative fluorescent density of fluorescent images analyzed using ZEN software (V1.0.1.0, Carl Zeiss, Oberkochen, Germany) was presented as values relative to the control sample. Fifty individual samples were randomly chosen and analyzed for each treatment.

### 2.10. Determination of TBARS and H_2_O_2_ Contents

Thiobarbituric acid-reactive substances (TBARS) and H_2_O_2_ contents were determined with spectrophotography [27,28]. For TBARS detection, the fresh sample tissues (0.2 g) were ground with 5% (*w/v*) trichloroacetic acid (TCA) on ice, and then 0.5% 2-thiobarbituric acid (TBA) was added to the 5% TCA. After incubation at 90 °C for 20 min, the absorbance of the sample was determined spectrophotometrically at 450, 532, and 600 nm. The TBARS amount was obtained using an extinction coefficient of 157 mM^−1^ cm^−1^ and expressed as nmol g^−1^ fresh weight.

For H_2_O_2_ detection, the assay reagent was added to an aliquot of supernatant. After 45 min of incubation, the absorbance of the Fe^3+^–xylenol–orange complex was determined at 560 nm. The H_2_O_2_ content was detected by using an extinction coefficient of 0.28 μM^−1^ cm^−1^ and expressed as μmol g^−1^ fresh weight.

### 2.11. Analysis of Antioxidant Enzyme Activity

Fresh samples (0.5 g) were homogenized in cooled phosphate buffer (pH 7.8). After centrifugation, the supernatant was used to determine the activities of antioxidant enzymes. The ascorbate peroxidase (APX), catalase (CAT), guaiacol peroxidase (POD), superoxide dismutase (SOD), and glutathione reductase (GR) activities of the seedlings were determined as previously described [29,30]. One enzyme unit (U) of SOD was considered to be the amount of enzyme corresponding to 50% inhibition of NBT reduction.

The monodehydroascorbate reductase (MDHAR) and dehydroascorbate reductase (DHAR) activities of the seedlings were determined with an MDHAR Kit and DHAR Kit (Sinobestbio, Shanghai, China).

### 2.12. qPCR Analysis

Total RNA was isolated with a TransZol Up Kit (TransGen Biotech, Beijing, China). After total RNA from whole plants was extracted and the synthesis of cDNA analyzed, quantitative real-time PCR (qPCR) was performed. Primer sequences for qPCR are supplied in Supplementary Table S1. The relative-fold expression was calculated by using the 2^−ΔΔCT^ method with *MSC27* and *Actin2* as the two reference genes [31].

### 2.13. Statistical Analysis

All experiments had three independent biological replicates. Values were shown as the means ± standard deviation (SD). Statistical significance was determined using one-way analysis of variance (ANOVA) followed by Duncan’s multiple range test (*p* < 0.05).

## 3. Results

### 3.1. Improvements in Seed Germination and Root Growth Inhibition Achieved by Argon

**Figure 1:**
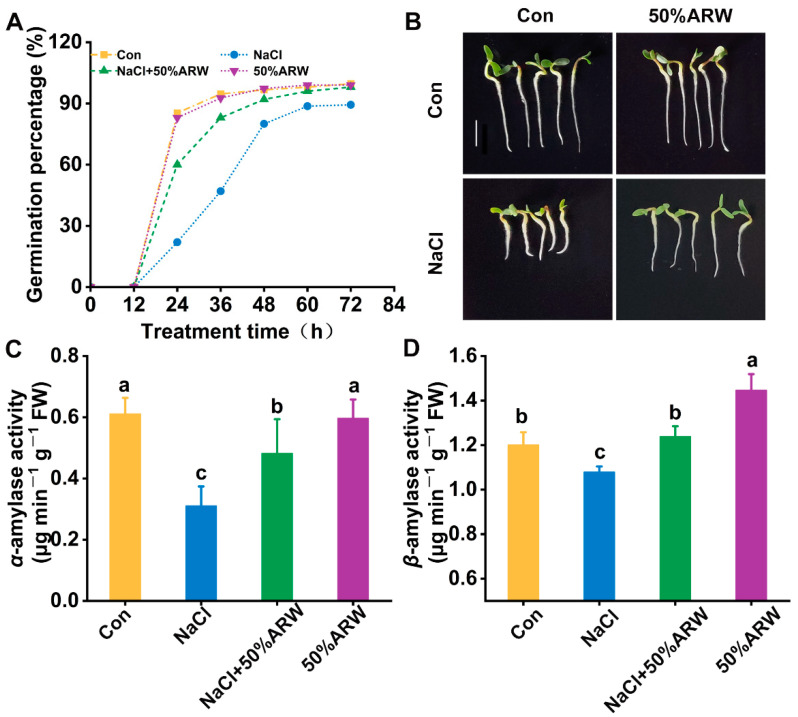
The inhibition of seed germination and reduction in amylase activity caused by NaCl stress were alleviated by argon. Alfalfa seeds were incubated in 0 (Con) or 50% argon−rich water (ARW) with or without 100 mM NaCl. Germination percentage was recorded at the indicated time points (**A**). After treatments for 72 h, phenotypic photos were taken (**B**). Bar = 1 cm. The activities of *α*-amylase (**C**) and *β*-amylase (**D**) were also detected. The error bars represent the SD (*n* = 3; 100 samples/treatment/repeat for (**A**)). Bars with different letters (a–c) were significantly different at *p* < 0.05 according to Duncan’s multiple range test.

In laboratory conditions, the optimal concentration of argon to apply to NaCl-stressed alfalfa during seed germination was determined in the presence of argon-rich water (ARW) with various saturations of 10%, 25%, 50%, and 100% (Table S2). As expected, after NaCl stress for 36 h and 72 h, significant decreases in germination percentage and root length were observed in the stressed samples compared to the NaCl-free control. In contrast, the above inhibition was differentially abolished by ARW addition. Fifty percent saturated ARW was subsequently selected, since this concentration, compared to the non-stress controls, was found to remarkably alleviate the inhibitory impacts resulting from NaCl stress on germination percentage (−11.9 ± 2.0% vs. −50.3 ± 4.0% for 36 h; −3.0 ± 1.0% vs. −10.0 ± 2.6% for 72 h) and root length (−28.6 ± 3.2% vs. −48.4 ± 4.1% for 72 h) in an approximately time-dependent fashion (Figure 1A,B). Consistently, the decreases in both *α*-amylase (especially) and *β*-amylase activities caused by salinity stress were obviously reversed in the presence of 50% ARW (72 h; Figure 1C,D). We also noticed that the treatment with ARW alone for 72 h did not influence germination percentage and root growth under the non-stressed conditions.

To confirm the specific function of argon, nitrogen gas-rich water (NRW) was also used. It was clearly observed that, unlike the beneficial role of ARW, NRW failed to address the cytotoxic effects resulting from NaCl stress in alfalfa seed germination (Table S3). These results preliminarily ruled out the involvement of the hypoxia effect in ARW addition.

### 3.2. Ion Homeostasis Was Reestablished by Argon

**Figure 2:**
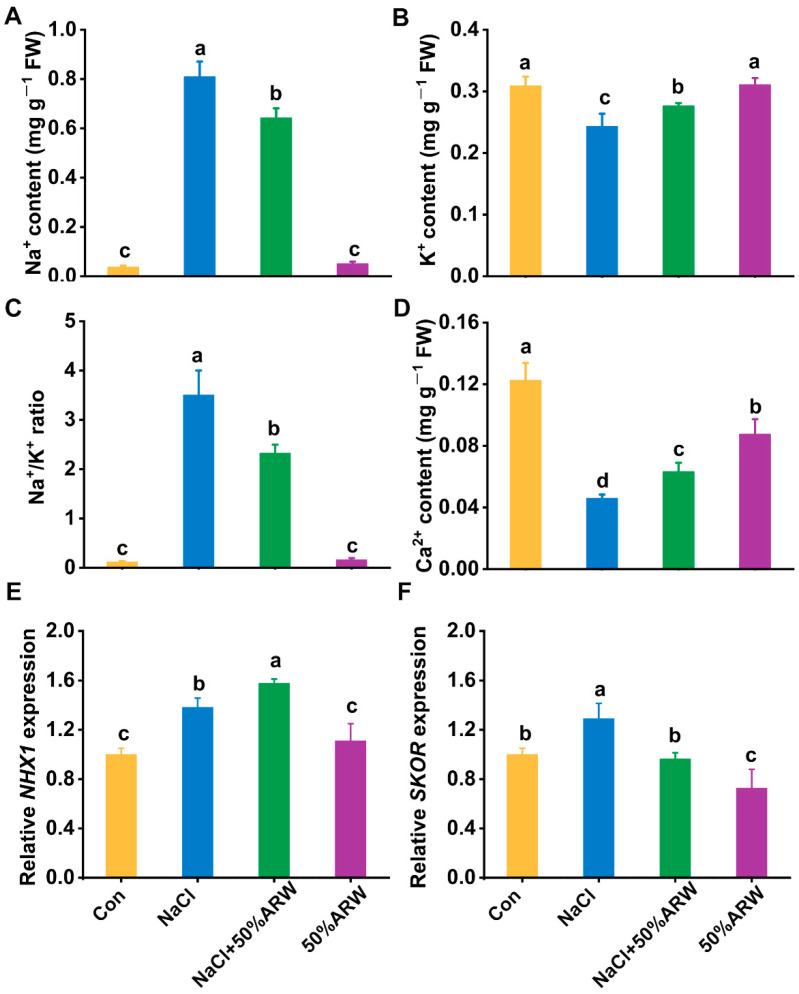
Changes in ion homeostasis in response to argon. After germination for 3 d, the Na^+^ (**A**) and K^+^ (**B**) contents in the seedlings, as well as the Na^+^/K^+^ ratio (**C**), were determined. The Ca^2+^ content (**D**) was also calculated. Changes in *NHX1* (**E**) and *SKOR* (**F**) transcripts in seedlings were analyzed with qPCR. The error bars represent the SD. Bars with different letters (a–d) were significantly different at *p* < 0.05 according to Duncan’s multiple range test.

Compared with alfalfa seedlings under non stressed conditions, NaCl stress resulted in a significant increase in Na^+^ content from about 0.036 ± 0.007 to 0.808 ± 0.063 mg g^−1^ FW and an obvious reduction in K^+^ content from 0.308 ± 0.015 to 0.242 ± 0.021 mg g^−1^ FW, thus remarkably increasing Na^+^/K^+^ (Figure 2A–C). Meanwhile, the Ca^2+^ content decreased to 39.6% that of the non-stressed level (Con) after NaCl treatment (Figure 2D).

Since reestablished ion homeostasis plays an important role in plant tolerance against salinity stress, we further examined the effects of ARW on the ion contents and element ratio. As shown in Figure 2A, ARW significantly reduced the over-accumulation of Na^+^ content caused by NaCl stress. This addition also had an obvious inducing effect on the K^+^ content, and the ratio of Na^+^/K^+^ was significantly decreased (Figure 2B,C). The reduction in Ca^2+^ content resulting from the NaCl stress was partly abolished in the presence of argon (Figure 2D).

Subsequently, we examined the transcripts responsible for Na^+^ compartmentation and K^+^ exclusion. The transcriptional profiles of NHX1 (encoding the vacuolar Na^+^/H^+^ antiporter [32]) and SKOR (responsible for root-to-shoot translocation of K^+^ [33]) in the seedlings were up-regulated by NaCl stress, and each was respectively intensified or impaired by argon (Figure 2E,F). The above results clearly suggested that ion homeostasis was reestablished by argon.

**Figure 3:**
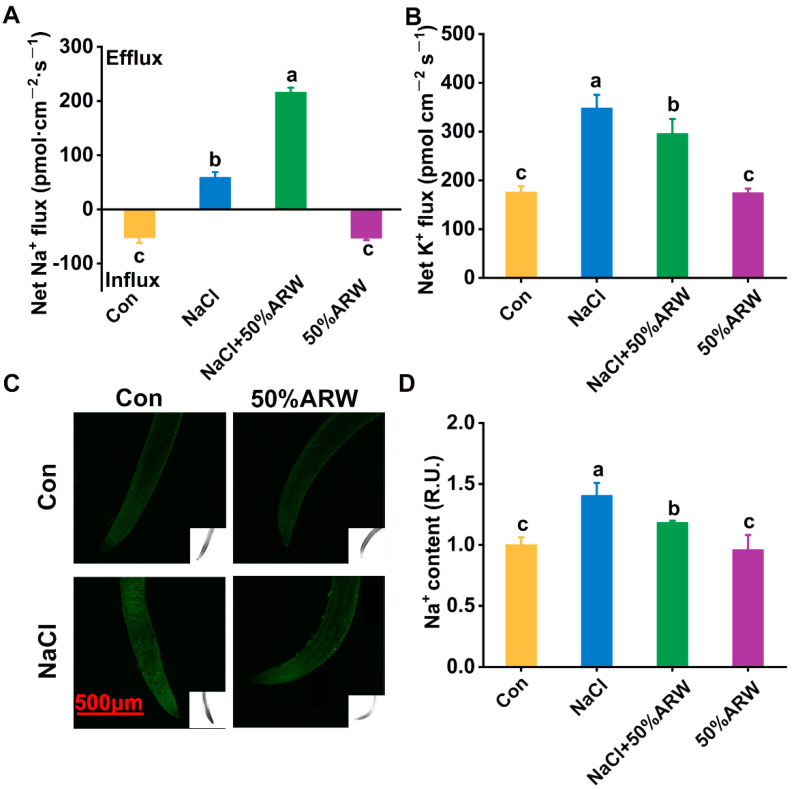
Ion homeostasis was reestablished by argon. After germination for 3 d, net Na^+^ (**A**) and K^+^ fluxes (**B**) in the mature root zone were monitored. Meanwhile, Na^+^ fluorescence (**C**) in the root tips was also determined. Mean relative Na^+^ fluorescence intensities (**D**) corresponding to (**C**) are given. R.U., relative unit. Bar = 500 μm. The error bars represent the SD (*n* = 3; 10–50 samples/treatment/repeat). Bars with different letters (a–c) were significantly different at *p* < 0.05 according to Duncan’s multiple range test.

To further elucidate the molecular mechanism, non-invasive micro-test technology (NMT) was applied. As shown in Figure 3A, the NaCl stress-triggered net Na^+^ efflux in the roots of the alfalfa seedlings was intensified by argon. Under similar conditions, the increased net K^+^ efflux caused by salinity was abolished by argon, helping to keep more K^+^ in the root tissues (Figure 3B).

Subsequently, the Na^+^ levels in the alfalfa seedling root tissues were monitored using fluorescence microscopy [34]. As expected, NaCl stress-triggered fluorescence intensity in the root tips was impaired when ARW was added (Figure 3C,D). These results further reflected the fact that argon could reestablish ion homeostasis upon NaCl stress.

### 3.3. Argon-Treated Plants Decreased Oxidative Damage Resulting from NaCl Stress

**Figure 4:**
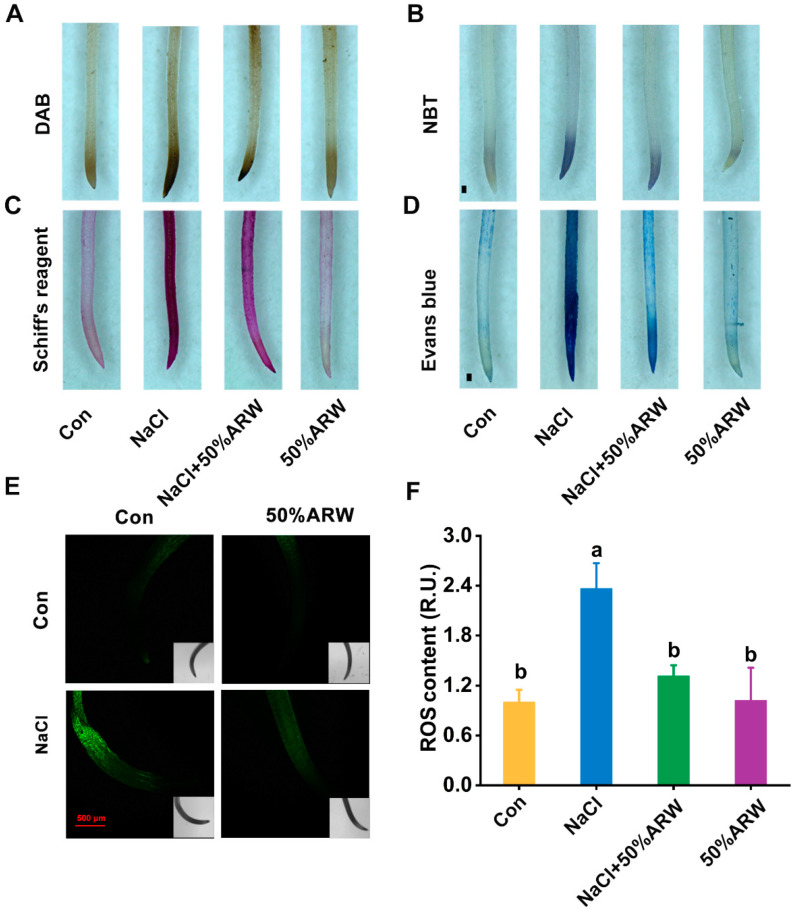
Redox homeostasis was reestablished by argon. After germination for 3 d, root tissues were stained with DAB (**A**) and NBT (**B**) to visualize the distribution of H_2_O_2_ or O_2_^−^, respectively. The roots were stained with Schiff’s reagent (**C**) and Evans blue (**D**) to determine lipid peroxidation and plasma membrane integrity, respectively. Bars = 0.5 mm. DCF-dependent fluorescence (**E**) was also imaged, and relative intensities (**F**) are given. R.U., relative unit. Bar = 500 μm. The error bars represent the SD (*n* = 3; 50 samples/treatment/repeat). Bars with different letters (a,b) were significantly different at *p* < 0.05 according to Duncan’s multiple range test.

To probe the mechanism underlying argon’s control of tolerance against NaCl stress, the distribution of endogenous H_2_O_2_ and O_2_**^●^**^−^ was assessed. Compared to the basal levels, NaCl stress resulted in some dark brown (DAB staining) and purple-blue (NBT staining) color precipitates (Figure 4A,B), confirming greater ROS accumulation upon stress. In contrast, the addition of ARW could differentially weaken the above staining, showing that argon could suppress H_2_O_2_ and O_2_**^●^**^−^ accumulation.

The above changes in ROS led us to assess oxidative damage. We examined lipid oxidation and plasma membrane integrity using histochemical staining. Consistent with the profiles for the ROS levels (Figure 4A,B), argon treatment was found to abolish the increased staining patterns resulting from NaCl stress (Figure 4C,D). These results were further supported by the changes in the ROS contents determined with laser confocal analysis (Figure 4E,F). Thus, our findings further revealed a unique regulatory role for argon in the regulation of the homeostasis of redox in alfalfa seedling roots.

**Figure 5:**
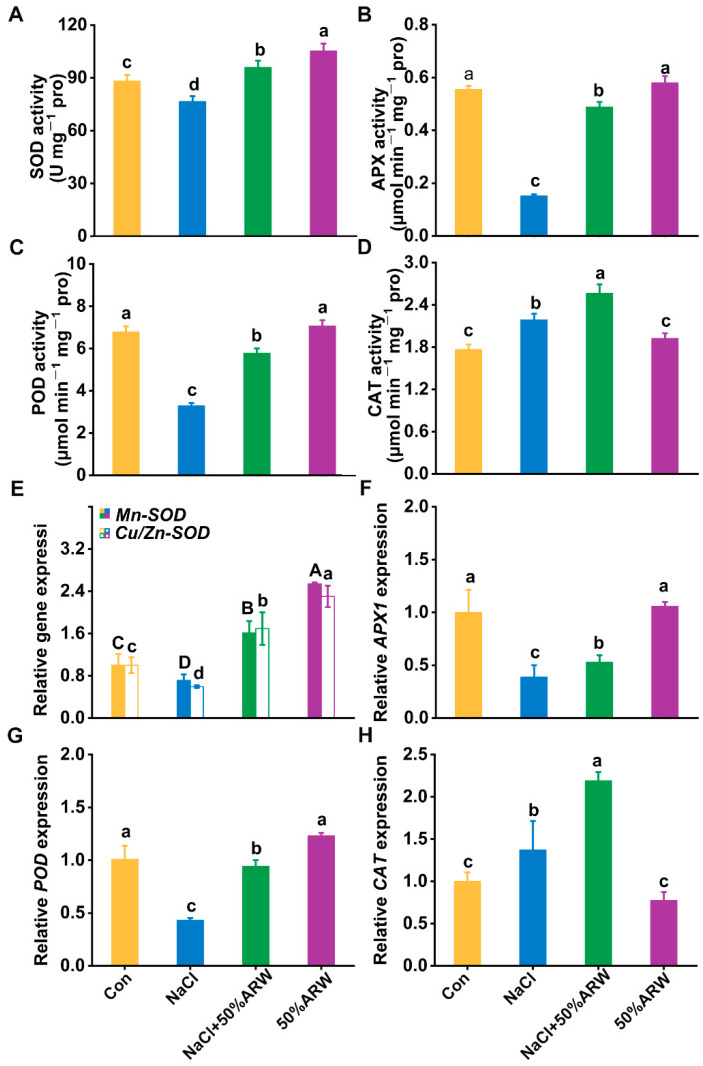
Changes in H_2_O_2_-related antioxidant enzyme activities and transcripts in alfalfa plants. After germination for 3 d, the activities of SOD (**A**), APX (**B**), POD (**C**), and CAT (**D**) were determined. Transcripts of Mn-SOD/Cu/Zn-SOD (**E**), APX1 (**F**), POD (**G**), and CAT (**H**) were analyzed by qPCR. The error bars represent the SD. Bars with different letters were significantly different at *p* < 0.05 according to Duncan’s multiple range test.

**Figure 6:**
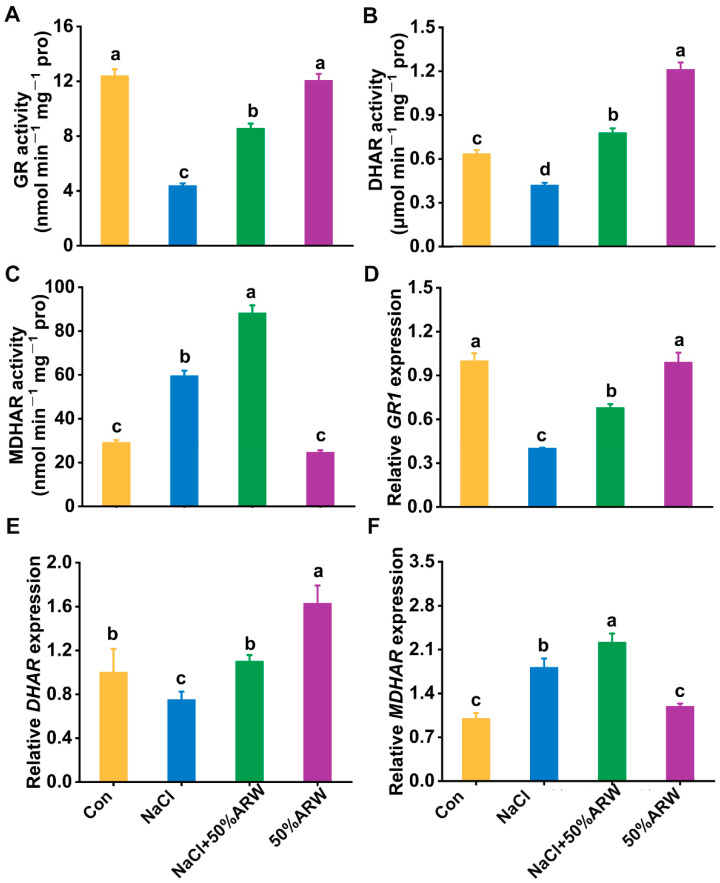
Changes in AsA-GSH-related antioxidant enzyme activities and transcripts in alfalfa plants. After germination for 3 d, the activities of GR (**A**), DHAR (**B**), and MDHAR (**C**) were determined. Transcripts of *GR1* (**D**), *DHAR* (**E**), and *MDHAR* (**F**) were analyzed by qPCR. See Table S1 for detailed gene expression information. The error bars represent the SD (*n* = 3). Bars with different letters were significantly different at *p* < 0.05 according to Duncan’s multiple range test.

Antioxidant enzymes play essential roles in antioxidant defense; thus, the activities of several antioxidative enzymes were investigated. Subsequent results showed that the activities of superoxide dismutase (SOD), ascorbate peroxidase (APX), guaiacol peroxidase (POD), glutathione reductase (GR), and dehydroascorbate reductase (DHAR) in the alfalfa seedlings decreased after NaCl stress for 72 h, being 13.2, 72.8, 51.6, 64.7, and 33.8% lower than those of the control samples, respectively (Figure 5A–C and Figure 6A,B). In contrast, the application of ARW modified the reduction (*p* < 0.05) in these five antioxidant enzymes activities. Moreover, significant increases in catalase (CAT) and monodehydroascorbate reductase (MDHAR) were observed in the presence of NaCl, which were further strengthened by argon (Figure 5D and Figure 6C).

Similarly, NaCl stress resulted in decreases in the transcript levels of *Mn-SOD*, *Cu/Zn-SOD*, *APX1*, *POD*, *GR1*, and *DHAR* (Figure 5E–G and Figure 6D,E), all of which were obviously blocked after incubation with ARW. Comparing the enzymatic activity changes, similar tendencies were observed in *CAT* and *MDHAR* transcripts (Figure 5H and Figure 6F).

### 3.4. Pot Experiments Confirmed Argon Functioning in the Regulation of Salinity Tolerance

**Figure 7:**
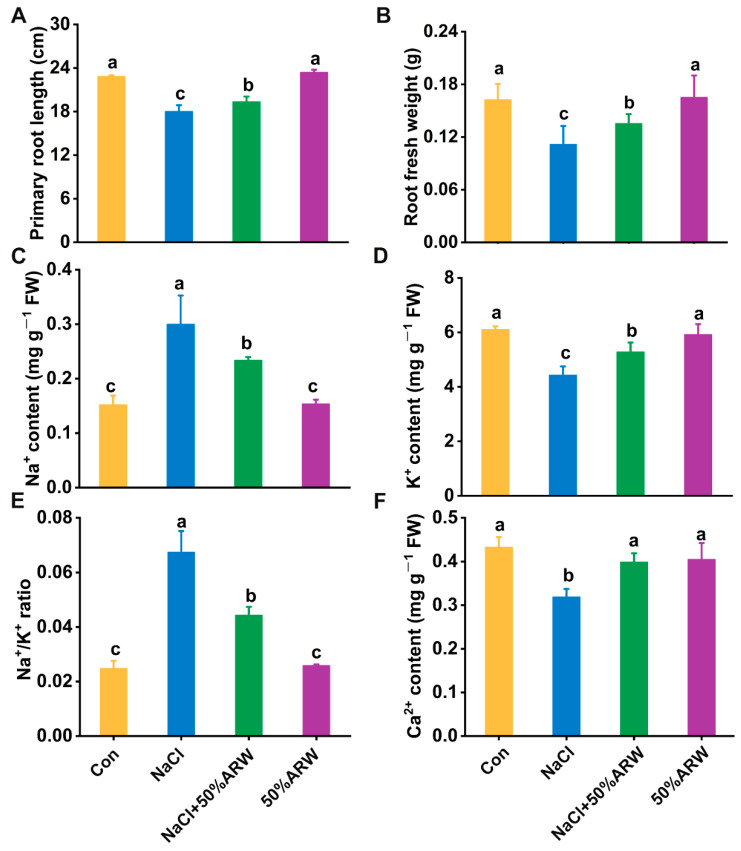
Inhibition of alfalfa growth and ion imbalance caused by NaCl stress were improved by argon. After 21 days of the pot treatment, the root length (**A**) and fresh weight (**B**) were measured (*n* = 3; 27 samples/treatment/repeat). Additionally, the Na^+^ (**C**) and K^+^ (**D**) contents in the root tissue, as well as the Na^+^/K^+^ ratio (**E**) and the Ca^2+^ contents (**F**), were determined and calculated. The error bars represent the SD. Bars with different letters were significantly different at *p* < 0.05 according to Duncan’s multiple range test.

In order to confirm argon functioning in the regulation of salinity tolerance in plants, a pot experiment involving culturing in a greenhouse was adopted. Our experiments confirmed that the physiological phenotypic performance of the three-week-old alfalfa seedlings upon NaCl stress was obviously improved by the 50% ARW. After irrigation for another 21 days, the better performance facilitated by argon was clearly observed, as determined by changes in the primary root growth and the fresh weight of root tissues (Figure 7A,B).

To evaluate the effect of argon on ion homeostasis in the roots of the six-week-old alfalfa plants, changes in the ion contents and the element ratio were assessed. Similarly to the changes during the seed germinating stage, exposure of the alfalfa plants in potting soil upon NaCl stress brought about a marked increase in the Na^+^ content and decreases in the K^+^ and Ca^2+^ contents, thus resulting in an increase in the Na^+^ to K^+^ ratio in the root tissues (Figure 7C–F). The above changes were differentially impaired by irrigation with ARW, reflecting the reestablishment of ion homeostasis achieved by argon.

**Figure 8:**
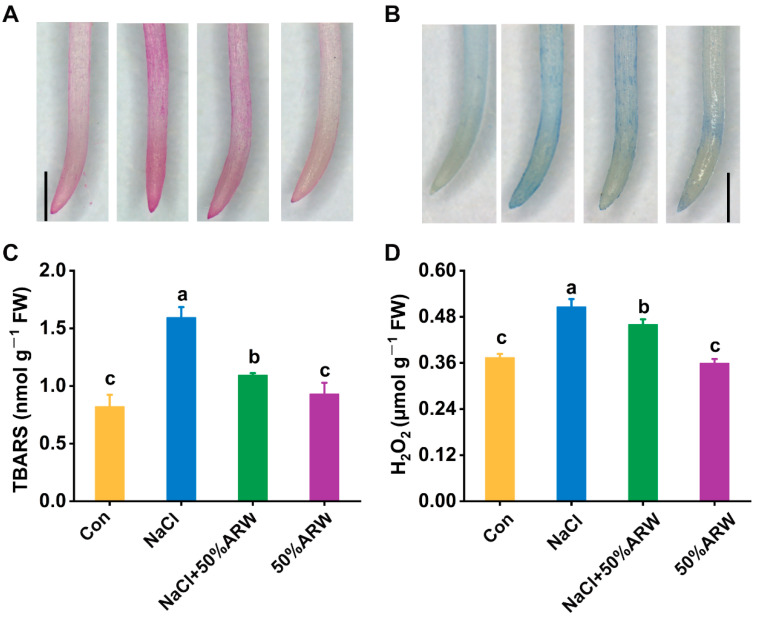
Argon alleviates NaCl-induced oxidative damage. After 21 days of pot treatment, the roots were stained with Schiff’s reagent (**A**) and Evans blue (**B**) to determine lipid peroxidation and plasma membrane integrity, respectively. Bars = 1 mm. The contents of TBARS(**C**) and H_2_O_2_ (**D**) in root tissues were analyzed. The error bars represent the SD. Bars with different letters were significantly different at *p* < 0.05 according to Duncan’s multiple range test.

Further experiments clearly showed that argon could alleviate lipid peroxidation and the loss in plasma membrane integrity in six-week-old alfalfa plants upon salinity, since the heavy staining with both Schiff’s reagent and Evans blue resulting from NaCl stress could be clearly attenuated after irrigation with ARW (Figure 8A,B). Changes in TBARS and H_2_O_2_ contents displayed similar tendencies (Figure 8C,D).

## 4. Discussion

Work in recent decades has led to an understanding that argon can act as a putative functioning molecule in animals [35,36,37]. However, information about whether or how argon influences salt stress response in plants is still elusive. In this work, we demonstrated that argon positively improved salt tolerance in laboratory and greenhouse conditions at the alfalfa seed germination and seedling growth periods by reestablishing ion and redox balance. These are new findings. Since argon is relatively cheap and easy to apply—and, in particular, does not show any signs of adverse effects in cells [8]—our discovery is beneficial for both fundamental and applied plant biology.

First, we provide evidence of the beneficial effect of argon (in the form of ARW) on NaCl-induced phytotoxicity in alfalfa plants during seed germination (Table S2) and seedling growth (Figure 7A,B). Salinity stress severely influences several physiological processes during seed germination, such as imbibition, germination and root elongation, and seedling growth. Under our experimental conditions, the seed germination inhibition and stunted seedling growth elicited by salinity were clearly reversed by applying ARW with 50% saturation, which was further supported by the increased activities of *α*-amylase and *β*-amylase during seed germination compared to NaCl stress alone (Figure 1). The pot experiment also showed that, in the ARW-treated alfalfa plants, the changes in the primary root length and fresh weight of roots were more pronounced under salt-stress conditions—showing 4.02% and 21.12% increases, respectively—compared to those in the stress-alone plants (Figure 7A,B). However, the mitigating effects of argon were relatively weaker or disappeared at low or high saturations, especially for root growth (Table S2), reflecting the possibility that the protective action of argon may be restricted to a narrow concentration. A similar phenomenon was observed in the homeostasis of nitric oxide (NO) [38], a ubiquitous gaseous signaling molecule functioning in both animals and plants [39].

How does argon fulfill the above biological function at both the physiological and molecular levels in plants? It is well-known that maintaining the ion balance, and especially maintaining the Na^+^/K^+^ ratio, is very important for plants to survive under salinity stress [1]. Our findings reveal a previously unidentified modulation of ion homeostasis from argon. A close relation between argon and the element ratio was found in alfalfa seedlings. For example, the NaCl-increased Na^+^ content and the Na^+^/K^+^ ratio were obviously attenuated by the application of ARW (Figure 2A,C), indicating that ion homeostasis was reestablished. Ca^2+^ absorption was also stimulated by argon under salinity conditions (Figure 2D). This was an interesting finding since an increased Ca^2+^ level could enhance plant tolerance against salinity stress [40].

For the seed germination stage, the evaluation of net ion fluxes clearly illustrated that, compared with NaCl stress alone, greater Na^+^ efflux and lower K^+^ efflux appeared after the addition of argon (Figure 3A,B), which resulted in the decreased Na^+^ accumulation (Figure 3C,D). Meanwhile, the stimulation of *NHX1* transcript (Figure 2E) could be clearly observed, suggesting the potential regulatory function of argon in Na^+^ sequestration via the Na^+^/H^+^ exchanger protein. Since constitutive overexpression of *NHX1* confers Na^+^ accumulation in vacuoles and, as a result, salinity tolerance in plants has been previously confirmed [41], we speculated that, besides Na^+^ exclusion, Na^+^ compartmentation might also play a vital role in argon-facilitated salinity tolerance. Moreover, the lower K^+^ efflux elicited by argon can be partly explained by the down-regulation of *SKOR* mRNA in seedlings (Figure 2F), which has been found to be involved in K^+^ translocation to the shoots [33], thus resulting in K^+^ accumulation (Figure 2B) and plant tolerance against salinity stress (Table S2, Figure 1A,B). Similar results were confirmed in the pot experiment (Figure 5E–H).

The neuroprotective and anti-apoptosis roles of argon in animals have been linked to its control of the antioxidative property [8,9]. In plants, overproduction of ROS with exposure to salinity stress is usually caused by impaired electron transport processes in the chloroplasts, mitochondria, and plasma membrane [42]. Genetic experiments have confirmed that salt tolerance could be obtained by overexpression of some antioxidant genes, including *APX* [43] and *SOD* [44]. Here, it was further observed that argon could remarkably repress ROS accumulation during seed germination resulting from NaCl stress (Figure 4A,B,E,F), thus leading to the abrogation of oxidative damage in root tissues (Figure 4C,D). Similar results were observed in the pot experiment (Figure 8). The above results are consistent with argon’s action in animal models [8,45]. Two lines of evidence supported the antioxidative role of argon in plants. First, argon could increase the activities of SOD, APX, POD, and CAT in NaCl-stressed plants (Figure 5A–D), which can be explained by changes in the transcriptional profiles of *Mn-SOD*/*Cu*/*Zn-SOD*, *APX1*, *POD*, and *CAT* (Figure 5E–H). Second, similar results were found for the changes to the ascorbic acid (AsA)-glutathione (GSH)-related antioxidant system (Figure 6).

Ultimately, we concluded that the beneficial function of argon in plants might be attributable to the stimulation of antioxidant defense, which causally resulted in reestablishment of redox homeostasis. Considering the functional parallels between the neuroprotective and anti-apoptosis factors in animals and the plant responses against salinity stress achieved by argon, we further deduced that a conserved molecular mechanism might exist.

This research aims to prove the concept that a “noble gas is not inert always”, which could also expand the scope of gasotransmitters. This research will be helpful in understanding the functions of argon biology and could shed new light on argon-based agriculture. Furthermore, argon did not show any signs of adverse effects, at least under our experimental conditions. Importantly, argon is common in the atmosphere and relatively cheap, as well as easy to apply in low-carbon agriculture, compared to other agrochemicals.

## 5. Conclusions

Collectively, the results of this study demonstrate the positive role of argon in plant salinity tolerance in the stages of both seed germination and seedling growth. The molecular mechanism can be explained by the reestablishment of ion and redox homeostasis, two important issues for plant tolerance against salinity. To our knowledge, this is the first report to address the functional role of argon in plant physiology, and these findings might open a new window for the study of argon biology in plant kingdoms. Furthermore, this research will help in applying argon in agriculture. Argon gas, as a non-polar molecule, easily permeates into tissue, where it then possibly interacts with mostly amphiphilic proteins. Thus, some direct interaction between argon and proteins may exist, which could be a crucial point for elucidating argon biological effects in the future. Additionally, the argon signal transduction pathway should be considered, as well as the crosstalk with other well-known gasotransmitters.

## Data Availability

Data are contained within the article and supplementary files.

## Supplementary Materials

The following supporting information can be downloaded at: https://www.mdpi.com/article/10.3390/antiox11061168/s1, Table S1: The sequences of primers for qPCR; Table S2: Effects of argon-rich water (ARW) on germination parameters upon NaCl stress; Table S3: Effect of nitrogen-rich water (NRW) on germination percentage upon NaCl stress.
