# Supplementary material for: A New Discovery of Argon Functioning in Plants: Regulation of Salinity Tolerance
Source: Antioxidants (Basel). 2022 Jun 14;11(6):1168. doi: 10.3390/antiox11061168 (PMC9220380; doi:10.3390/antiox11061168)
Supplement: Supplementary file 1 [file antioxidants-11-01168-s001.zip › antioxidants-1758764-supplementary.pdf]

Supporting Information

This file includes:

Supplementary materials and methods

Table: S1-S3

Pages: 4

Supplementary Table S1: The sequences of primers for qPCR

Primer name	*M. truncatula* tentative consensus or Accession Number	Sequences
GR1	AM407890	Forward: TGTGTCATTCGTGGTTGTG<br>Reverse: ACCCGCTATCTTTCCCTC
MDHAR	JN979555	Forward: GTCAAACTAAAGGACGGAAGGGTA<br>Reverse: AGCAACATCGCCAACAGCAT
DHAR	DQ006811	Forward: AATGACGGAACTGAACAA<br>Reverse: TACTTTAGGACGCCAACC
Cu/Zn-SOD	AF056621	Forward: TAATTGCTGATGCCAACG<br>Reverse: ACCACAGGCTAATCTTCCAC
Mn-SOD	AY145894	Forward: TGTCATCAGCGGCGTAATCAT<br>Reverse: GGGCTTCCTTTGGTGGTTCA
APX1	DQ122791	Forward: TCCTCTTATGCTCCGTTTG<br>Reverse: GTTCCACCCAGTAATCCCA
POD	X90695	Forward: TTTGTCATTGGCAGGTGAT<br>Reverse: TGAAACTTGGCTGAGGGA
CAT	TC100988	Forward: TTCTTCTTCTCCACCGTCCTCA<br>Reverse: TCCAAGAGAATTGGACCTCTGG
NHX1	XM_003591016.4	Forward: ATAATCTTCCACGGCAGCTCAA<br>Reverse: TCTCATCAACACAAGTATCTCTCG
SKOR	XM_003616199	Forward: TGATGGCGATCTGTACCAGC<br>Reverse: TCAAGTAGCGGTGTGTTCCC
MSC27	X63872	Forward: AGAATGGAATGTTGTGGGAGG<br>Reverse: GTCATCAACACCCTCATCTTCTC
Actin2	JQ028730	Forward: AAAAGGATGCCTATGTTGGTG<br>Reverse: AAGTGGAGCCTCAGTTAGAAGTA

Supplementary Table S2 Effects of argon-rich water (ARW) on germination parameters upon NaCl stress

Treatments	Germination percentage (%; 36 h)	Germination percentage (%; 72 h)	Root length (mm; 72 h)
Con	94.67±3.05a	99.33±0.57a	27.63±0.34a
10%ARW	87.67±2.51b	97.67±1.52a	27.23±0.92a
25%ARW	90.00±2.00b	98.33±1.53a	27.52±0.90a
50%ARW	93.67±0.57a	99.00±1.00a	28.35±1.46a
100%ARW	86.67±1.15bc	98.00±2.00a	27.74±0.72a
NaCl	47.00±2.64f	89.33±2.31c	14.25±0.34c
NaCl+10%ARW	78.67±3.05e	92.67±1.15b	15.38±1.01c
NaCl+25%ARW	80.33±1.52de	92.67±3.05b	15.71±1.42c
NaCl+50%ARW	83.33±1.73cd	96.33±2.30a	19.72±0.37b
NaCl+100%ARW	82.33±2.08de	93.00±2.64b	15.67±1.32c

Germination percentage and root length were recorded at the indicated time points. The error bars represent the SD (n = 3; 100 samples/treatment/repeat). All experiments have three independent biological replicates. Values were shown as the means ± standard deviation (SD). Statistical significance was determined using one-way analysis of variance (ANOVA) followed by Duncan's multiple range test ($P < 0.05$).

Supplementary Table S3 Effect of nitrogen-rich water (NRW) on germination percentage upon NaCl stress

Treatments	Germination percentage (%; 36 h)	Germination percentage (%; 72 h)
Con	92.67±3.06a	97.33±2.08a
10%NRW	90.00±2.00a	96.67±3.21a
25%NRW	92.00±3.47a	97.33±1.15a
50%NRW	96.00±0.00a	97.67±1.52a
100%NRW	89.33±4.62a	97.00±2.64a
NaCl	51.33±8.33bc	89.00±2.64b
NaCl+10%NRW	47.00±3.00bc	86.00±2.00b
NaCl+25%NRW	55.33±3.05b	90.67±3.05b
NaCl+50%NRW	56.67±9.45b	87.33±4.61b
NaCl+100%NRW	42.00±8.72c	88.67±2.31b

Germination percentage was recorded at the indicated time points. The error bars represent the SD (n = 3; 100 samples/treatment/repeat). All experiments have three independent biological replicates. Values were shown as the means ± standard deviation (SD). Statistical significance was determined using one-way analysis of variance (ANOVA) followed by Duncan's multiple range test ($P < 0.05$).
